# Associations of psychotic symptom dimensions with clinical and developmental variables in twin and general clinical samples

**DOI:** 10.1192/bjp.2024.129

**Published:** 2025-01

**Authors:** Alastair G. Cardno, Judith Allardyce, Steven C. Bakker, Timothea Toulopoulou, Eugenia Kravariti, Marco M. Picchioni, Fergus Kane, Frühling V. Rijsdijk, Tariq Mahmood, Soumaya Nasser el din, Deline du Toit, Lisa A. Jones, Diego Quattrone, James T. R. Walters, Sophie E. Legge, Peter A. Holmans, Robin M. Murray, Evangelos Vassos

**Affiliations:** Division of Psychological and Social Medicine, University of Leeds, Leeds, UK; Centre for Clinical Brain Sciences, University of Edinburgh, Edinburgh, UK; Centre for Neuropsychiatric Genetics and Genomics, Cardiff University, Cardiff, UK; Department of Psychosis Studies, Institute of Psychiatry, Psychology and Neuroscience, King's College London, London, UK; Department of Psychology and National Magnetic Resonance Research Center (UMRAM), Bilkent University, Ankara, Turkey; Department of Psychiatry, National and Kapodistrian University of Athens, Athens, Greece; Department of Psychiatry, Icahn School of Medicine at Mount Sinai, New York, USA; Department of Forensic and Neurodevelopmental Science, Institute of Psychiatry, Psychology and Neuroscience, King's College London, London, UK; Department of Clinical Educational and Health Psychology, University College London, London, UK; Psychology Department, Anton de Kom University of Suriname, Paramaribo, Suriname; Social, Genetic and Developmental Psychiatry (SGDP) Centre, Institute of Psychiatry, Psychology and Neuroscience, King's College London, London, UK; Becklin Centre, Leeds & York Partnership NHS Foundation Trust, Leeds, UK; Cygnet Healthcare, Bradford, UK; Three Counties Medical School, University of Worcester, Worcester, UK

**Keywords:** Psychosis, symptoms, twins, familial, intelligence

## Abstract

**Background:**

Positive, negative and disorganised psychotic symptom dimensions are associated with clinical and developmental variables, but differing definitions complicate interpretation. Additionally, some variables have had little investigation.

**Aims:**

To investigate associations of psychotic symptom dimensions with clinical and developmental variables, and familial aggregation of symptom dimensions, in multiple samples employing the same definitions.

**Method:**

We investigated associations between lifetime symptom dimensions and clinical and developmental variables in two twin and two general psychosis samples. Dimension symptom scores and most other variables were from the Operational Criteria Checklist. We used logistic regression in generalised linear mixed models for combined sample analysis (*n* = 875 probands). We also investigated correlations of dimensions within monozygotic (MZ) twin pairs concordant for psychosis (*n* = 96 pairs).

**Results:**

Higher symptom scores on all three dimensions were associated with poor premorbid social adjustment, never marrying/cohabiting and earlier age at onset, and with a chronic course, most strongly for the negative dimension. The positive dimension was also associated with Black and minority ethnicity and lifetime cannabis use; the negative dimension with male gender; and the disorganised dimension with gradual onset, lower premorbid IQ and substantial within twin-pair correlation. In secondary analysis, disorganised symptoms in MZ twin probands were associated with lower premorbid IQ in their co-twins.

**Conclusions:**

These results confirm associations that dimensions share in common and strengthen the evidence for distinct associations of co-occurring positive symptoms with ethnic minority status, negative symptoms with male gender and disorganised symptoms with substantial familial influences, which may overlap with influences on premorbid IQ.

In psychosis, broadly defined psychotic symptoms show a pattern of co-occurrence that can be summarised by factor analysis into three main symptom dimensions: positive (hallucinations and delusions), negative (restricted speech and affect, reduced motivation and socialisation) and disorganised (formal thought disorder, incongruous/inappropriate affect and bizarre behaviour).^1–3^ In some studies, subdivisions or combined dimensions can occur, depending on which symptoms are included;^4,5^ affective symptoms^6–8^ or cognitive measures^9^ are sometimes also included in analyses.

## Associations with clinical and developmental variables

Among individuals with psychotic disorders, symptoms that tend to co-occur may share some risk factors and clinical consequences in common, which may be indicated by associations with the relevant symptom dimension. For all three symptom dimensions, there are well-established associations between higher factor or symptom scores (where core symptoms co-occur) and poor premorbid adjustment/functioning,^7,10–16^ and also chronic illness course, most strongly for the negative dimension.^10,12,17^ The positive dimension is also associated with cannabis use^18–20^ and the negative and disorganised dimensions with earlier age at onset.^7,8,10,11,13,21^

In addition, there are less consistent or less investigated associations, including for the positive dimension with later age at onset^11^ and ethnic minority status,^8^ for the negative dimension with male gender^8,10,22^ and obstetric complications^14^ and for the disorganised dimension with ethnic minority status,^23^ abnormal infant development^21^ and lower premorbid IQ.^7^

Interpretation of the above studies is complicated by different definitions of symptom dimensions and clinical and developmental variables. This includes differences in rating scales and approaches to factor or symptom scores. In addition, some variables have undergone relatively little investigation. It would therefore be valuable to investigate further using the same definitions across multiple samples.

## Familial/genetic influences

Among sibling pairs with schizophrenia and other psychoses, each of the three psychotic symptom dimensions shows modest familial aggregation (within-pair correlations up to *r* ~ 0.3), with most consistent results for the disorganised dimension.^4,24–26^

In a study of monozygotic (MZ) twin pairs concordant for psychosis, correlations for the positive and negative dimensions were similar to those for sib-pairs. However, for the disorganised dimension there was a higher correlation (*r* ~ 0.7)^27^ and substantive twin heritability (~80%).^28^

It would be valuable to build on these findings by investigating familial aggregation of symptom dimensions in a further independent sample of twins with psychosis.

## Study aims

This study aimed to investigate associations of psychotic symptom dimensions with clinical and developmental variables, and the degree of familial aggregation of symptom dimensions, in multiple samples that employed the same definitions of symptom dimensions and other variables assessed on a lifetime basis.

## Method

### Samples

The study was based on four UK mental health service psychosis samples: two twin samples and two general clinical samples.

The register twin sample was systematically ascertained from the Maudsley Twin Register^29^ in London and assessed by interview^30,31^ and case record review.

The non-register twin sample was based on the combined Maudsley schizophrenia and bipolar twin study samples,^32,33^ recruited nationally and assessed by interview^31,34,35^ and case record review. There was no overlap with the register twin sample.

The Clinical Variation in Psychoses Study (CVPS) was recruited in West Yorkshire, England, and assessed by interview^35^ and case record review.

The Dumfries and Galloway Psychosis Study (D&G)^13,36^ was recruited systematically in south-west Scotland and assessed by case record review.

Further information is given in Supplementary Methods and Results and Supplementary Tables, including concordance for schizophrenia and psychosis, and zygosity distributions in the twin samples (Supplementary Tables 1 and 2 available at https://doi.org/10.1192/bjp.2024.129).

The Maudsley twin register was established before the Declaration of Helsinki and research ethics committees but was based on consistent principles. The non-register twin study had approval from the UK Multicentre National Health Service (NHS) Research Ethics Committee and the Ethics Committee of the Institute of Psychiatry, Psychology and Neuroscience (IoPPN), King's College London. All participants gave written informed consent. The CVPS had NHS Research Ethics Committee approval and all participants gave written informed consent. The D&G had NHS Research Ethics Committee approval. Written informed consent was not required as it was a service-based case record review study.

Table 1 shows the characteristics of the four samples in order of illness duration. The register twin sample probands had the longest illness duration (mean 21.7 years (s.d. 12.6)) and highest prevalence of psychotic symptoms, broadly defined to include positive, negative and disorganised symptoms. The non-register twin probands had the narrowest range of diagnoses, predominantly comprising individuals with schizophrenia or bipolar disorder.

**Table 1:** Characteristics of the four psychosis samples

Characteristic	Twin sample (register)	Twin sample (non-register)	CVPS general clinical sample	D&G general clinical sample
Ascertainment	Systematic	Non-systematic	Non-systematic	Systematic
No. probands	223	123	76	456
% male	53.4	50.4	80.3	44.5
% White ethnicity	87.9	96.6	82.9	100
Distribution of main-lifetime diagnoses (%)	DSM-III-R Schizophrenia 43.5 RDC Schizophrenia 47.5 Schizoaffective 14.3 Mania 12.1 Other affective disorder 10.8 Other psychoses 15.2	DSM-IV Schizophrenia 57.7 Schizoaffective 1.6 Bipolar I 39.0 Bipolar II 1.6	DSM-IV Schizophrenia 38.2 Schizoaffective 11.8 Bipolar I 36.8 Other affective disorder 2.6 Other psychoses 10.6	DSM-IV Schizophrenia 24.3 Schizophreniform 2.6 Schizoaffective 3.5 Bipolar I 9.6 Other affective disorder 11.6 Delusional disorder 39.9 Other psychoses 8.3
Year of onset (first contact with mental health services) (range)	1948–1993	1960–2002	1990–2010^a^	1979–1998
Age at onset (first contact with mental health services) (mean, (s.d.), range)	26.3 (11.1) years	21.1 (6.3) years	26.5 (8.9) years	41.8 (20.2) years
	range 7–67 years	range 5–50 years	range 12–57 years	range 14–94 years
Illness duration (age at last information minus age at onset) (mean, (s.d.), range)	21.7 (12.6) years	16.4 (11.4) years	14.5 (12.8) years	12.4 (7.3) years
	range 0–60 years	range 1–49 years	range 0–44 years	range 0–26 years
Positive symptom dimension scores (score: *n* (%))	0: 9 (4.0)	0: 18 (14.6)	0: 7 (9.3)	0: 34 (7.5)
	1: 62 (27.8)	1: 24 (19.5)	1: 27 (36.0)	1: 176 (38.6)
	2: 152 (68.2)	2: 81 (65.9)	2: 41 (54.7)	2: 246 (53.9)
Negative symptom dimension scores (score: *n* (%))	0: 119 (53.4)	0: 70 (56.9)	0: 62 (82.7)	0: 342 (75.0)
	1: 69 (30.9)	1: 29 (23.6)	1: 11 (14.7)	1: 67 (14.7)
	2: 35 (15.7)	2: 24 (19.5)	2: 2 (2.7)	2: 47 (10.3)
Disorganised symptom dimension scores (score: *n* (%))	0: 102 (45.7)	0: 88 (71.5)	0: 56 (74.7)	0: 265 (58.1)
	1: 70 (31.4)	1: 28 (22.8)	1: 16 (21.3)	1: 123 (27.0)
	2: 51 (22.9)	2: 7 (5.7)	2: 3 (4.0)	2: 68 (14.9)

CVPS, Clinical Variation in Psychoses Study; D&G, Dumfries and Galloway Psychosis Study; RDC, research diagnostic criteria.

a. Inferred from period of recruitment and illness duration.

The CVPS had the highest proportion of males (80.3%) owing to recruitment including patients from male in-patient wards, and the highest proportion of Black and minority ethnicity people (17.1%). The D&G had the broadest range of age at onset (14–94 years). It also had a lower prevalence of schizophrenia and higher prevalence of delusional disorder than the other samples.

### Study variables

#### Symptom variables

Psychotic symptom dimensions were defined as symptom scores based on Operational Criteria Checklist (OPCRIT)^37^ lifetime-ever symptoms. They comprised the most consistent core symptoms from previous factor analyses^1–4,9^ and have been used in previous studies of symptom dimensions.^4,27,28^ They were as follows.

(a) Positive dimension (Pos) (scored 0–2): hallucinations (score 1) and delusions/thought interference/passivity (score 1).

(b) Negative dimension (Neg) (scored 0–2): negative formal thought disorder (i.e. poverty of speech) (score 1) and restricted/blunted affect (score 1).

(c) Disorganised dimension (Dis) (scored 0–2): positive formal thought disorder (score 1) and inappropriate affect (score 1).

Further rationale and details are given in Supplementary Methods and Results and Supplementary Tables, including correlations between symptom dimensions (Supplementary Table 3) and correlations between symptom dimensions and affective symptoms (Supplementary Table 4).

In some samples few participants scored 0 for Pos or 2 for Neg and Dis (Table 1), so for the main analyses the symptom dimensions were dichotomised into Narrow and Broad forms. The main focus was on the Narrow form, where participants had to have both symptoms present (score 0–1 *v*. 2), for example, both hallucinations and delusions for the positive dimension. There was sufficient symptom variation to also analyse the Broad form for Neg and Dis, where participants had to have at least one symptom present (score 0 *v*. 1–2). Inclusion of the Broad form allowed observation of patterns of association in samples where there were relatively few scores of 2 for Neg and Dis.

#### Clinical and developmental variables

The clinical and developmental variables were as follows. Most were based on lifetime ratings of the OPCRIT checklist:^37^ gender, ethnicity, poor premorbid social adjustment, never married/cohabited, premorbid substance misuse, lifetime cannabis misuse/recurrent use, presence of a psychosocial precipitant, age at onset, rate of onset, illness course, birth order within twin pair, birthweight, handedness and premorbid IQ (based on the National Adult Reading Test (NART)^38^).

Further details are given in Supplementary Methods.

### Analysis

#### Associations between symptom dimensions and clinical and developmental variables

We conducted logistic regression analyses with presence/absence of the Narrow symptom dimension as the dependent variable and the clinical or developmental variable as the independent variable, adjusted for gender and illness duration. In analyses of the combined samples we used a generalised linear mixed model with the addition of sample modelled as a random effect to account for possible sample effects. We also conducted analysis of presence/absence of the Broad symptom dimension for Neg and Dis.

In the twin samples we restricted these analyses to twin probands.

We conducted an additional investigation of the independence of Narrow symptom dimension associations in the combined samples, by adding the other two dimensions as covariates in each analysis: for example, in the logistic regression analysis of Narrow Pos on clinical and developmental variables, the covariates were gender, illness duration, Narrow Neg and Narrow Dis. If a symptom dimension association found in the main analysis became non-significant, this could indicate that the association is at least partly dependent on co-occurrence with symptoms of other correlated dimensions.

Further information about testing logistic regression models is given in Supplementary Methods and Results.

Variables were described as associated where *P* < 0.05, two-tailed. To account for multiple statistical testing, we used the Benjamini–Hochberg false discovery rate (FDR = 0.05) as the primary threshold for statistical significance for the main results (Table 2).

**Table 2:** Logistic regression analysis of narrow psychotic symptom dimensions on demographic, developmental and clinical variables among probands in the combined samples^a^

Independent variable	*n*	Positive dimension	Negative dimension	Disorganised dimension
		Odds ratio (95% CI)	Odds ratio (95% CI)	Odds ratio (95% CI)
Male gender [this analysis not adjusted for gender]	875	1.18 (0.90–1.56)	2.02 (1.32–3.09)**	1.33 (0.90–1.95)
Black and minority ethnicity [D&G excluded as all White ethnicity]	412	3.07 (1.30–7.25)**	1.22 (0.49–3.06)	0.80 (0.31–2.10)
Poor premorbid social adjustment	875	1.99 (1.44–2.74)**	2.41 (1.58–3.68)**	2.57 (1.73–3.81)**
Never married or cohabited	875	1.85 (1.39–2.45)**	2.86 (1.81–4.53)**	2.03 (1.36–3.03)**
Premorbid drug/alcohol misuse (within year before onset)	875	1.48 (1.00–2.19)	0.95 (0.53–1.71)	1.35 (0.81–2.26)
Lifetime cannabis misuse/regular use	875	1.89 (1.19–3.00)**	1.39 (0.74–2.61)	1.92 (1.08–3.40)*
Psychosocial precipitant (within 6 months before onset)	875	0.75 (0.54–1.05)	0.67 (0.38–1.18)	0.93 (0.59–1.49)
Age at onset (first contact with mental health services)	875	0.98 (0.97–0.99)**	0.94 (0.92–0.96)**	0.94 (0.92–0.96)**
Gradual onset (over more than 6 months)	733	1.11 (0.81–1.52)	1.89 (1.19–3.01)**	2.05 (1.38–3.05)**
Chronic illness course	842	2.41 (1.76–3.31)**	9.06 (5.52–14.89)**	2.95 (1.99–4.37)**
Birth order (being second born in twin pair) [twins only]	252	0.58 (0.32–1.04)	0.75 (0.41–1.40)	1.29 (0.69–2.39)
Birthweight (kg) [D&G excluded as no information]	188	1.08 (0.68–1.74)	1.11 (0.68–1.81)	1.51 (0.87–2.61)
Handedness (being non-right handed) [D&G excluded as no information]	209	1.65 (0.62–4.43)	1.86 (0.65–5.32)	1.97 (0.80–4.88)
Premorbid IQ (NART) [twins only]	83	0.98 (0.93–1.03)	0.96 (0.92–1.00)*	0.92 (0.88–0.98)**

D&G, Dumfries and Galloway Psychosis Study; NART, National Adult Reading Test.

a. Combined sample analysis used generalised linear mixed model, adjusted for gender and illness duration with sample modelled as a random effect.

Statistically significant at *P < 0.05, two-tailed and **after adjustment for multiple statistical testing using Benjamini–Hochberg false discovery rate (FDR = 0.05).

Analyses were conducted in SPSS version 29 for Mac (Cupertino, CA, USA; see https://www.ibm.com/spss).

#### Familial aggregation of psychotic symptom dimensions

In the twin samples we calculated tetrachoric correlations (*r*_tet_) for the Narrow and Broad symptom dimensions in twin pairs concordant for psychosis. We focused on correlations for MZ pairs as there were relatively few concordant dizygotic (DZ) pairs (*n* = 13 in the samples combined) so confidence intervals were wide. We did not make heritability estimates because the non-register twin sample was not systematically ascertained.

We regarded 95% confidence intervals not overlapping zero as consistent with a statistically significant correlation at *P* < 0.05, two-tailed.

Analyses were conducted in OpenMx for Mac (Cupertino, CA, USA; see https://openmx.ssri.psu.edu).

## Results

### Associations with clinical and developmental variables

Results for the Narrow symptom dimensions in the combined samples are summarised in Table 2. Further details including results for individual samples and Broad symptom dimensions are in Supplementary Table 5. Descriptive statistics for each variable across symptom dimension levels are in Supplementary Table 6. Results for the Narrow symptom dimension associations independent of the other dimensions are in Supplementary Table 7.

In the combined samples, all three Narrow dimensions were associated with poor premorbid social adjustment, never marrying/cohabiting and earlier age at onset, including when conditioning on the other dimensions. Results were similar for the Broad dimensions, and were consistent across individual samples, except that Pos was not associated with never marrying/cohabiting in D&G.

All three dimensions were associated with a chronic course, but effect sizes were greater for Neg than for the other dimensions (Narrow Neg odds ratio = 9.06 (95% CI: 5.52–14.89), Broad Neg odds ratio = 5.74 (95% CI: 4.10–8.05)). Effect directions were consistent across samples. Overall, among individuals with a chronic course, 28.3% had both negative symptoms (Neg score = 2), compared with 4.3% of individuals who did not have a chronic course.

Pos was associated with Black and minority ethnicity overall and in the register twins and CVPS. D&G was not included in this analysis as all participants were of White ethnicity. Overall, among individuals of Black and minority ethnicity, 84.1% had both delusions and hallucinations (Pos score = 2), compared with 62.2% of individuals of White ethnicity.

Pos was associated with lifetime cannabis misuse/regular use overall and in D&G. Dis was also associated but not after adjustment for multiple statistical testing. Overall, among individuals with lifetime cannabis misuse/regular use, 70.1% had both delusions and hallucinations (Pos score = 2), compared to 57.8% of individuals without cannabis misuse/regular use.

Neg was associated with male gender, with consistent results across Narrow and Broad definitions, and individual samples except for the register twins. CVPS had too little variation to be analysed individually because all 12 participants with negative symptoms were male. Overall, among males, 15.5% had both restricted affect and poverty of speech (Neg score = 2), compared with 9.0% of females.

There were no associations between symptom dimensions and birthweight in the combined samples, but there was evidence of a non-linear association for Narrow Neg in the register twins only. (Further details are given in Supplementary Methods and Results.)

Narrow Dis was associated with gradual illness onset overall and in the register twins and D&G. Neg was also associated but not independently of the other symptom dimensions. Pos was also associated in two individual samples, but not overall because of the lack of association in D&G. Overall, among individuals with gradual onset, 22.9% had both formal thought disorder and inappropriate affect (Dis score = 2), compared to 12.2% of individuals with a more rapid onset.

Dis was associated with lower premorbid IQ. Caveats include that only the twin samples had premorbid IQ data, and only a subgroup of twins in each sample, so sample size was relatively small (*n* = 83 twin probands overall) and confidence interval wider. However, odds ratios were similar across Narrow and Broad definitions and across the two twin samples. Narrow Neg was also associated but not after adjustment for multiple statistical testing. Overall, mean premorbid IQ was 11.8 points lower in twins with both disorganised symptoms compared to twins with neither of the disorganised symptoms (mean IQ 97.9 *v.* 109.7).

### Familial aggregation of symptom dimensions

Tetrachoric correlations for symptom dimensions in MZ twin pairs concordant for psychosis are shown in Table 3. Pos had a significant correlation of modest size (*r*_tet_ = 0.33 (95% CI: 0.01–0.60)). Narrow Neg had a non-significant correlation of modest size (*r*_tet_ = 0.26 (95% CI: −0.16 to 0.62)). There was a significant correlation for Broad Neg, but for associations with symptom dimensions per se we would expect correlations for the Narrow definition to be similar to or greater than for the Broad definition. The significant correlation for Broad Neg but not Narrow Neg could reflect familial aggregation for an absence of negative symptoms related to reduced emotional expression. Narrow Dis had a significant and more substantial correlation (*r*_tet_ = 0.74 (95% CI: 0.41–0.91)), with consistent results across Narrow and Broad definitions and the two twin samples.

**Table 3:** Tetrachoric correlations of psychotic symptom dimensions within monozygotic (MZ) twin pairs concordant for any psychotic disorder

Sample		Positive dimension	Negative dimension		Disorganised dimension	
		Narrow (0–1 *v*. 2)	Broad (0 *v*. 1–2)	Narrow (0–1 *v*. 2)	Broad (0 *v*. 1–2)	Narrow (0–1 *v*. 2)
	No. pairs	*r*_tet_ (95% CI)	*r*_tet_ (95% CI)	*r*_tet_ (95% CI)	*r*_tet_ (95% CI)	*r*_tet_ (95% CI)
Twin (register)	46	0.22 (−0.25 to 0.62)	0.39 (−0.05 to 0.72)	0.32 (−0.40 to 0.83)	0.81 (0.50–0.95)^a^	0.63 (0.16–0.90)^a^
Twin (non-reg.)	50	0.42 (−0.01 to 0.74)	0.64 (0.27–0.86)^a^	0.18 (−0.34 to 0.64)	0.45 (−0.02 to 0.78)	0.84 (0.28–0.99)^a^
Combined	96	0.33 (0.01–0.60)^a^	0.53 (0.25–0.74)^a^	0.26 (−0.16 to 0.62)	0.72 (0.49–0.87)^a^	0.74 (0.41–0.91)^a^

*r*_tet_, tetrachoric correlation coefficient.

a. Correlations with 95% CI not including zero treated as statistically significant at *P* < 0.05, two-tailed.

### Secondary analysis

As Dis was associated with substantial familial aggregation and lower premorbid IQ we conducted secondary analyses to further investigate the relationship between these factors. First, we investigated the degree of familial aggregation for premorbid IQ in MZ twin pairs concordant for psychosis and found this to be substantial (intraclass correlation (ICC) = 0.79 (95% CI: 0.58–0.90): Supplementary Table 8). Caveats include the small number of twin pairs where both members had premorbid IQ data (*n* = 24 pairs overall). However, correlations were significant in both twin samples individually as well as combined.

Second, we investigated evidence for familial influences shared in common between Dis and premorbid IQ, by investigating associations between Dis in twin probands and premorbid IQ in their co-twins, using the same logistic regression analysis approach as for the main investigation of clinical and developmental variables. Results are shown in Supplementary Table 9. In MZ twin pairs, there was an association between the presence of Narrow Dis in probands and lower premorbid IQ in their co-twins (*n* = 55; odds ratio = 0.84 (95% CI: 0.71–0.99); *P* = 0.039), with similar results for the Broad definition and consistent trends in both individual twin samples. In DZ twin pairs, there was no association (for Narrow Dis, odds ratio = 1.03 (95% CI: 0.87–1.21)) but interpretation is limited by the small number of DZ pairs (*n* = 21 overall).

## Discussion

Higher symptom scores on all three symptom dimensions were associated with poor premorbid social adjustment, never marrying/cohabiting and earlier age at onset. Previous studies have also found all three dimensions to be associated with poor premorbid social adjustment,^7,10–16^ for Neg to be associated with being single^10^ and for Neg and Dis to be associated with earlier age at onset.^7,8,10,11,13,21^ A previous association of positive symptoms with later age at onset^11^ was not confirmed in this study.

All three dimensions were associated with a chronic course, but more strongly for Neg than the other dimensions, which is consistent with previous studies.^10,12,17^

Pos was associated with lifetime cannabis misuse/regular use, which is consistent with previous studies.^18–20^ Further information including age at first use and frequency and strength of cannabis used is likely to give additional insights into the relationship, owing to evidence for cumulative dosage effects.^18,19^

Pos was associated with Black and minority ethnicity, which has been previously reported.^8^ This may be because of the enduring stresses of being in a minority ethnic group in the UK.^39^ There is a caveat that clinicians/researchers with insufficient cultural understanding may mis-label some culturally congruent beliefs as positive symptoms.^40^ While some mis-labelling cannot be excluded, the association was most notable in CVPS, where clinicians/researchers were often from the relevant ethnic group themselves. Black and minority ethnicity includes a broad range of ethnic/cultural groups. In the register twins, African/Caribbean ethnicities were most common, and in CVPS, South Asian ethnicities, both of which are still broad groupings. The association may occur relatively widely across minority ethnic groups, but this requires further investigation.

Neg was associated with male gender, which has been found in some studies^8,10,22^ but not in others.^6,11^ The current findings strengthen the evidence for an association, but the reasons for this need clarification. Consistent with primary developmental factors being involved, high negative symptom scores in adolescent twins have been associated with male gender, along with substantive heritability, cumulative pre-/perinatal complications and lower childhood cognitive functioning;^41^ consistent with secondary consequences of having psychosis being involved, Neg and male gender were strongly associated in D&G, where negative symptoms during the first year of onset were uncommon.^13^ However, the interaction between gender and risk factors for negative symptoms needs further investigation.

Previous association of Neg with obstetric complications^14^ was not confirmed in the combined samples, at least as indexed by birth order in twins and birthweight. Associations of small effect size or with other pre-/perinatal variables cannot be excluded and, as noted above, associations between negative symptoms and cumulative pre-/perinatal complications have been found in a longitudinal study of adolescent twins.^41^ It would be valuable to investigate whether the non-linear association with birthweight in the register twins is found in other samples.

Narrow Dis was associated with gradual onset. Neg was also associated but not independently of the other dimensions. Association with Neg has been previously reported.^10,12^ It would therefore be useful to investigate further the extent to which this is dependent on co-occurrence with disorganised symptoms.

Dis was associated with lower premorbid IQ. The analysis was confined to the twin samples, and only a minority of the twins had NART scores for analysis, so further confirmation is required. Also the lowest IQ in the sample was 70, so associations for IQs below this could not be assessed. An association between Dis and lower premorbid IQ has previously been observed in a general psychosis sample,^7^ but not consistently.^21^

Previous associations of Dis with presence of a psychosocial precipitant^12^ and ethnic minority status^23^ were not confirmed.

Regarding familial aggregation of symptom dimensions, Pos had a significant correlation of modest size (*r*_tet_ = 0.33) in MZ twin pairs concordant for psychosis. We were not able to directly compare with the DZ correlation, but the MZ correlation is similar to the correlation in affected sibling pairs with schizophrenia or schizoaffective disorder (*r*_polychoric_ = 0.36),^25^ and to the level of familial aggregation in other affected sibling pair samples.^24^ This is consistent with the contribution of some shared environmental influences to variation in positive symptoms among individuals with psychosis. A candidate in the current study is the stresses associated with ethnic minority status, and this could be investigated further in larger, more ethnically diverse samples; however, it would not explain the correlation in sib-pairs who were all of White ethnicity.^25^ A further candidate is factors linked to parental socioeconomic status (SES) but, against this, none of the dimensions is associated with parental SES in the register twin sample (*n* = 197 probands; *r*_s_ = −0.01–0.05).

The non-significant correlation for Narrow Neg is consistent with the non-significant correlation for Neg in the sib-pair study with the most similar phenotypic definition^25^ and the inconsistent results for correlations in other sib-pair studies.^24^ There is other evidence for genetic influences on negative symptoms, for example, associations with schizophrenia polygenic risk score (PRS),^26,42^ and the variation in results for Neg between studies could be because of differences in phenotype definitions and which risk factors predominate.

Dis had a significant and more substantial correlation (*r*_tet_ = 0.74) in MZ pairs concordant for psychosis. This had been previously found for the register twins,^27^ and was further substantiated in the current study with the addition of the independent non-register twin sample. Investigation in additional samples would be valuable where feasible, to reduce the width of confidence intervals around estimates. The correlation for Dis in MZ pairs is considerably larger than in affected sib-pairs (*r*_polychoric_ = 0.25),^25^ and familial aggregation in other sib-pair samples (*r* ~ 0.3),^24^ and consistent with the substantial heritability for Dis found in the register twin sample (~80%).^28^ We did not extend the investigation of heritability in the current study because of the non-systematic ascertainment of the non-register twins. Evidence for genetic influences on Dis is also emerging from analyses of schizophrenia PRS.^5,9,42^

Finally, in secondary analysis, we found evidence of substantial familial aggregation for premorbid IQ (MZ pair ICC = 0.79), consistent with previous twin/family studies of Wechsler Adult Intelligence Scale full-scale IQ that have included the non-register twins (MZ pair ICC = 0.81;^43^ *h*^2^ = 54–73%^32,33,43,44^). We also found preliminary evidence that Dis and premorbid IQ share some familial influences, which requires further confirmation. Statistical modelling in the register twins does not suggest substantial shared environmental influences on Dis,^28^ nor on IQ in studies that included the non-register twins.^32,33,43,44^ So if there are notable familial influences they may be mainly because of inherited genetic variants. In MZ twins, they could also be because of *de novo* variants that are present before the twins separate in utero. In terms of commonly occurring genetic variants, premorbid IQ is associated with IQ PRS,^45^ but Dis showed only a trend towards association with lower IQ PRS in a sample of individuals with schizophrenia or schizoaffective disorder.^9^ Premorbid IQ is also associated with rare coding schizophrenia risk alleles and copy number variants (CNVs),^45^ but to our knowledge these associations have not yet been reported for Dis.

### Strengths and limitations

To our knowledge, this is the first study to investigate psychotic symptom dimensions in multiple twin and general clinical samples, allowing the reporting of associations with risk factors and clinical variables in individuals and also familial influences.

The results could have differed if we had used different definitions of psychotic symptom dimensions, for example, based on factor scores, other rating instruments or including subdivisions of dimensions. However, using symptom scores facilitated interpretation and the definitions we used focused on the most consistent core symptoms for each dimension in previous factor analyses.^1–4,9^

The results could also have differed if we had used different clinical or developmental variable definitions, although all are well-established. Further risk factors could be investigated, for example, childhood trauma^46^ and urbanicity.^47^ The current samples had an urbanicity gradient of register twins > non-register twins and CVPS > D&G, but the degree of urban upbringing at an individual level was not assessed.

The findings of this study should be viewed in the context of clinical variation within lifetime psychotic disorders and may not necessarily extrapolate to populations of individuals with a specific psychotic disorder or to the general population.

In each sample there was usually sufficient time since onset for most symptoms to become evident, and two of the samples were systematically ascertained. However, the timing of onset of particular symptoms was not known and developmental variables were assessed retrospectively. Longitudinal cohort studies may be valuable to enhance these assessments and broaden the range of cognitive and other developmental variables.^41^

Focusing on symptom dimensions can be valuable, for example, for investigating associations that symptoms within a dimension may share in common, but may mask some associations that are with a specific symptom only. Therefore, further investigation of individual psychotic symptoms, including interaction with affective symptoms, would also be valuable.

Some associations of small effect size may have been missed because of limitations in sample size and interrater reliability, and some additional confounders/biases may not have been accounted for.

Finally, while some associations in this study are consistent with the results of multiple previous investigations, others require further confirmation.

### Implications

These results confirm previous findings regarding associations between psychotic symptom dimensions and social adjustment, age at onset, chronicity and cannabis use. They also notably strengthen the evidence that lifetime co-occurrence of core positive symptoms is associated with ethnic minority status, negative symptoms with male gender and disorganised symptoms with substantial familial influences, which may overlap with influences on premorbid IQ, providing a basis to inform future research.

If the current findings continue to be substantiated and further clarified, they may contribute to the broader process of using symptom dimensions to complement diagnoses in the assessment and management of patients, including when considering different symptom profiles among individuals with the same diagnosis, and similarities in symptoms across diagnoses. In some instances, focusing on individual symptoms may be most useful, for example, for psychological therapies that focus on specific delusions or hallucinations; in others focusing at the level of symptom dimensions may be useful, for example, regarding positive symptoms responding to antipsychotic medication or negative symptoms being associated with chronic illness course. In this context, improved understanding of the processes associated with particular symptoms and their co-occurrence may, for example, contribute to further development of the symptom qualifiers and dimensions in ICD-11^48^ and DSM-5-TR.^40^

## Supporting information

Cardno et al. supplementary material 1 Cardno et al. supplementary material

Cardno et al. supplementary material 2 Cardno et al. supplementary material

Cardno et al. supplementary material 3 Cardno et al. supplementary material

Cardno et al. supplementary material 4 Cardno et al. supplementary material

## Data Availability

The OpenMx R script for calculating the tetrachoric correlations was written by F.V.R. and is available on reasonable request via A.G.C. OPCRIT variable definitions and additional guidance, originally written by L.A.J., A.G.C. and colleagues for a previous symptom dimensions study,^4^ are available on reasonable request to A.G.C. Summary statistics for associations between symptom dimensions and clinical/developmental variables are in Supplementary Table 6. CVPS participants have consented for de-identified research data to be shared with independent researchers, which can be done on reasonable request to A.G.C. Any requests for other data sharing should be directed to A.G.C.
