# Supplementary material for: Associations of psychotic symptom dimensions with clinical and developmental variables in twin and general clinical samples
Source: Br J Psychiatry. 2025 Jan;226(1):16–23. doi: 10.1192/bjp.2024.129 (PMC11781858; doi:10.1192/bjp.2024.129)
Supplement: Cardno et al. supplementary material 1 — Cardno et al. supplementary material [file S0007125024001296sup001.docx]

**Associations of psychotic symptom dimensions with clinical and developmental variables in twin and general clinical samples**

Cardno AG, et al.

**Supplementary Methods and Results**

**Samples**

Further information:

The register twin sample was systematically-ascertained from the Maudsley Twin Register. Probands comprised all individuals of multiple birth who attended any facility of the Maudsley and Bethlem Royal Hospitals in London between 1948 and 1993, and had psychotic symptoms or a manic/hypomanic episode. There were 223 probands and 224 probandwise pairs – the additional pair was from a triplet proband paired with their two co-triplets. Study assessments were based on clinical research interviews and review of case records by research psychiatrists or psychologists.

The non-register twin sample was based on the combined Maudsley schizophrenia and bipolar twin study samples. Probands with schizophrenia or bipolar disorder and their co-twins were recruited nationally through the UK National Health Service by referrals from treating psychiatrists and through voluntary support groups. It was not systematically ascertained. Individuals with schizophrenia who had harmful substance use or dependence in the past 12 months were excluded. There were 123 probands, 112 of whom had a recruited co-twin. Study assessments were based on clinical research interviews and review of case records by research psychiatrists or psychologists.

The Clinical Variation in Psychoses Study (CVPS) was based on a sample ascertained from mental health services in West Yorkshire, UK, between 2008 and 2013. It comprised 76 individuals, again with any psychotic symptoms or a manic/hypomanic episode. It was not systematically ascertained. Study assessments were based on clinical research interviews and review of case records by psychiatrists.

The Dumfries and Galloway Psychosis Study (D&G) was based on a systematically-ascertained incident sample comprising consecutive new referrals of individuals with psychotic disorders to mental health services in the Dumfries and Galloway region of south-west Scotland, between 1979 and 1998. This region is more rural than the locations of the other samples. Inclusion criteria were based on clinical ICD-9/ICD-10 diagnoses. Depressive psychosis was not included due to coding not differentiating between psychotic and non-psychotic depression. The original sample comprised 464 individuals, of whom 456 were followed up and given lifetime clinical research ratings. Study assessments were based on review of case records by a research psychiatrist (JA). A small number of participants also had clinical research interviews as a validation check.

**Description of OPCRIT**

The Operational Criteria Checklist (OPCRIT)^1^ is a checklist containing 90 items, for rating psychotic and affective symptoms (we used binary (present/absent) ratings for the current study) along with risk factors and illness history variables. It was designed to be rated from multiple sources including lifetime clinical research interviews and review of case records. OPCRIT has established reliability^1,2^ and is a convenient and widely used assessment tool. OPCRIT is agnostic regarding symptom dimensions, i.e. it was not created with particular symptom dimensions in mind.

In the current study lifetime ratings were made based on all relevant information from research interviews and clinical case records.

**Definitions of Psychotic Symptom Dimensions**

Positive dimension (Pos) (scored 0-2): hallucinations (score 1) and delusions/thought interference/passivity (score 1).

Hallucinations rated as present (score 1) if any of the OPCRIT items 64, 65, 72-77 rated as present.

Delusions/thought interference/passivity rated as present (score 1) if any of the OPCRIT items 54, 55, 57-71 rated as present.

Negative dimension (Neg) (scored 0-2): negative formal thought disorder (i.e. poverty of speech) (score 1) and restricted/blunted affect (score 1).

Negative formal thought disorder rated as present (score 1) if OPCRIT item 29 rated as present.

Restricted/blunted affect rated as present (score 1) if either OPCRIT item 32 or 33 rated as present.

Disorganised dimension (Dis) (scored 0-2): positive formal thought disorder (score 1) and inappropriate affect (score 1).

Positive formal thought disorder rated as present (score 1) if either OPCRIT item 27 or 28 rated as present.

Inappropriate affect rated as present (score 1) if OPCRIT item 34 rated as present.

Inter-rater reliability of symptom dimensions

Inter-rater reliability (mean r_s_ between raters for 30 cases) between the research psychiatrists and psychologist (including AGC and LAJ) who conducted ratings for the register twins was: Pos 0.69; Neg 0.65; Dis 0.66. Non-register twin ratings were conducted by research psychiatrists (EV, SB). This did not include formal assessment of inter-rater reliability but the raters frequently discussed cases and consulted with AGC. The CVPS did not include formal assessment of inter-rater reliability but ratings were conducted by AGC or by psychiatrists trained by him. All D&G ratings were conducted by a research psychiatrist (JA), who had high inter-rater reliability with a co-rater for OPCRIT variables in earlier analysis of this sample.^3,4^

Monozygotic (MZ) and dizygotic (DZ) twins in the two twin samples

Compared with the register twin sample, there was a higher proportion of MZ twins and higher concordance for schizophrenia in the non-register twin sample, but a similar concordance for psychosis (any psychotic disorder) (supplementary Table S1). Distributions of symptom dimension scores within the twin samples were similar in MZ and DZ twin probands (supplementary Table S2). In both twin samples, zygosity was assessed by similarity questionnaires and genetic markers.^5-7^

Correlations between symptom dimensions

There were positive correlations between symptom dimensions in each sample (supplementary Table S3).

Rationale for psychotic symptom dimension definitions

Psychotic symptom dimensions can also be based on, e.g. factor scores, which could retain more information and power. However, we found in previous analyses of familial aggregation in twins^8^ and siblings^9^ that the above OPCRIT symptom score definitions performed as well as OPCRIT or SAPS/SANS^10,11^ factor scores or more complex symptom scores, and are simpler to interpret.

Pos can have subdivisions but global hallucinations and delusions are the most consistent core symptoms for the positive dimension in factor analyses.^12-14^ The original studies have most commonly been based on SAPS/SANS global symptoms but we have translated the factor solutions into 0-2 OPCRIT symptom dimension scores and found similar levels of familiar aggregation, as described above.^9^ Neg can include symptoms related to reduced motivation/socialisation,^15^ but these are not available in OPCRIT and can be difficult to rate on a lifetime basis from interviews and case records. Dis can include bizarre behaviour, but we found in analysis of another sample that the confirmatory factor model fit was less good when this symptom was included.^15^

Factor analyses are also often based on PANSS,^16^ but we did not focus on these factor solutions as restricted/blunted and inappropriate affect are rated on the same variable, so negative and disorganised dimensions are less clearly separated.

We did not include affective symptoms in the analyses as these were assessed in different ways in different samples. However, it is unlikely that results were notably confounded by affective symptoms as higher scores on each of the psychotic symptom dimensions were associated with lower overall prominence of affective symptoms (supplementary Table S4).

**Definitions of Other Study Variables**

Definitions of study variables, other than symptom dimensions, were as follows. Most were based on lifetime ratings of the OPCRIT checklist^1^ and these are indicated.

(Inter-rater reliability in the register twin sample given in brackets: mean Spearman correlation or kappa coefficient between raters for 30 cases.)

Sex: female or male. OPCRIT variable.

Ethnicity: white or non-white. Best estimate based on interview and case record information.

Poor premorbid social adjustment (k=0.43): OPCRIT variable.

Patient found difficulty entering or maintaining normal social relationships, showed persistent social isolation, withdrawal or maintained solitary interests prior to onset of psychotic symptoms. (0,1)

Twins/CVPS additional guidance: If other relevant information is absent, a patient having had no friends at school or only one casual friend is rated ‘1’. Patients who had any more casual friends or any good friends are rated ‘0’.

Never married/cohabited (k=0.69): OPCRIT variable. (0,1)

Premorbid substance abuse (k=0.68): OPCRIT variable.

Alcohol/drug abuse within one year of onset of psychotic symptoms. Alcohol abuse where quantity is excessive (rater judgement) where alcohol related complications occur, during the year prior to first psychiatric contact (rated strictly as exclusion criteria for some definitions of schizophrenia). Drug abuse where non-prescribed drugs are repeatedly taken or prescribed drugs are used in excessive quantities and without medical supervision in year prior to first psychiatric contact. (0,1)

Twins/CVPS additional guidance: Excessive quantity of alcohol consumption is defined as greater than 21 units per week for males and greater than 14 units per week for females for at least one month. Alcohol related complications must be present and can be physical, social or psychological.

Lifetime cannabis abuse/regular use (k=0.64): OPCRIT variable.

Lifetime diagnosis of cannabis abuse/dependence. Continued use despite knowledge of having a persistent or recurrent social, occupational, psychological or physical problem that is caused or exacerbated by cannabis; or recurrent use in situations in which it is physically hazardous; or symptoms definitely indicative of dependence.

One of the above must have occurred persistently for at least one month, or repeatedly over a longer period. (0,1)

Twins/CVPS additional guidance: Regular drug use is defined as taking a drug at least once a week.

Presence of a psychosocial precipitant (k=0.54): OPCRIT variable.

Definite psychosocial stressor prior to onset. A severely or moderately severely threatening event has occurred prior to onset of disorder that is unlikely to have resulted from the subjects own behaviour. (ie the event can be seen as independent or uncontrollable). (0,1)

Twins/CVPS additional guidance: The patient must have experienced a psychosocial stressor within 6 months prior to illness onset. Stressors include: bereavement; serious illness; work problems; relationship problems; financial problems; and childbirth.

Age at onset (r_s_=0.92): OPCRIT variable.

Age of onset: This should be given to the nearest year and is defined as the earliest age at which medical advice was sought for psychiatric reasons or at which symptoms began to cause subjective distress or impair functioning. (enter age in years, eg 35)

Twins/CVPS additional guidance: First age at which medical advice is sought from mental health services for psychiatric problems.

Rate of onset (r_s_=0.47): OPCRIT variable.

Mode of onset

1= Abrupt onset definable to within hours or days

2= Acute onset definable to within 1 week

3= Moderately acute onset definable within 1 month

4= Gradual onset over period up to 6 months

5= Insidious onset over period greater than 6 months

Rate up if in any doubt.

Twins/CVPS additional guidance: A rating of ‘1’ is arbitrarily taken to mean onset definable within 3 days. Rate up unless there is no information about onset in which case an ‘m’ (missing) rating is appropriate.

As a score of 5 was the most common rating and gradations between other scores were often difficult to rate confidently, this variable was dichotomised to 1-4 (less gradual) vs 5 (gradual) in logistic regression analyses.

Illness course (r_s_=0.75): OPCRIT variable.

Course of disorder.

1= Single episode with good recovery

2= Multiple episodes with good recovery between

3= Multiple episodes with partial recovery between

4= Continuous chronic illness

5= Continuous chronic illness with deterioration

(NB score this item in hierarchical fashion, eg

if patient's course in past rated '2', but for the time-

period now being considered it rates '4', then the

correct rating is '4'.)

Twins/CVPS additional guidance:

Choosing between ratings of ‘4’ and ‘5’ - most patients with incapacitating chronic schizophrenia are rated as ‘4’ unless it is clear that they have gradually deteriorated in terms of severity of symptoms from the start of their illness to the present.

Choosing between ratings of ‘3’ and ‘4’ - if the patient appears to have had active positive psychotic symptoms or incapacitating symptoms for the majority of the illness duration, rate as ‘4’. If they have had these symptoms for the minority of the duration of the illness in episodes with residual symptoms between, they can be rated as ‘3’.

Rate ‘2’ if the patient resumes pre-morbid function between episodes.

Dichotomised to 1-3 (episodic) vs 4-5 (chronic) in logistic regression analyses.

Illness duration: age at last information minus age at onset.

*Further early developmental variables*

The following variables were assessed in smaller numbers of individuals or available in the twin samples only. They were based on retrospective information from interviews with twins, with a parent (including when also administering zygosity questionnaires), and from reviewing case records (or a specific test in the case of premorbid IQ).

Birth order within twin pair – an index of obstetric complications/perinatal morbidity as second born twin at higher risk of these.^17^

Birthweight (kg).

Handedness – right or left/ambidextrous.

Premorbid IQ – based on National Adult Reading Test (NART).^18^

For OPCRIT symptoms and other categorical variables, if there was no indication of these in the interviews or case records they were treated as absent. For other variables, analysis was conducted on those individuals where the relevant data was available. The numbers of individuals in each analysis are shown in the results tables.

**Previous Research Based on the Study Samples**

The register twin sample has been used to analyse familial/genetic influences,^5,19,20^ the non-register twin sample has been used for investigations of cognitive abilities,^6,7,21,22^ and the Dumfries and Galloway (D&G) sample has been used to report clinical associations occurring within a year of onset,^3^ but none of the samples has been used to investigate associations of lifetime symptom dimensions with clinical and developmental variables.

**Analysis**

The supplementary non-parametric regression analysis was conducted in R (https://cran.r-project.org); the other analyses were conducted in SPSS (https://www.ibm.com/spss).

Investigation of outliers

For each of the quantitative independent variables (illness duration, age at onset, birthweight and premorbid IQ), we investigated possible outlier observations using descriptive statistics including boxplots, where possible moderate outliers were flagged if they were more than 1.5 times the interquartile range (IQR) above the 3rd quartile or below the 1st quartile, and possible extreme outliers were flagged if more than 3 times the IQR.

When taking all participants in the four samples together, some of the longer illness duration observations in the twin samples, and some of the later age at onset observations in D&G, were flagged as possible moderate outliers (see also variable distributions in Table 1). However, we could find no evidence of rating errors, the possible outliers formed the tail end of their respective distributions rather than being separated from the other observations, and none of the observations were flagged as possible outliers within their respective samples, so we retained all of these observations in the analysis.

For illness duration paired with each of the other quantitative variables, we investigated possible bivariate outliers using the Mahalanobis distance between each data point and the centre of the (2-dimensional) distribution. Possible outliers were those observations whose Mahalanobis distance exceeded the p=0.001 criterion of a chi-squared distribution on 2df. The relatively stringent p-value threshold was because of the large number of statistical tests, one for each participant with each of the variables.

When taking all participants in the samples together, one duration-birthweight combination was flagged as a possible outlier in a register twin participant. However, it was not flagged as a possible outlier within the register twin sample, so was retained for analysis.

Investigation of multicollinearity

We investigated evidence for multicollinearity between independent variables using the Variance Inflation Factor (VIF), with VIF>10 indicating substantive multicollinearity and hence imprecise regression coefficients.

Taking all participants in the samples together, we found no evidence of substantive multicollinearity between independent variables (all VIFs<2).

Testing for linear associations in logistic regression models

For age at onset, birthweight, and premorbid IQ, we investigated the linearity of associations in logistic regression models using the Box-Tidwell test, where possible non-linearity is indicated by a significant association between the dependent variable (symptom dimension) with the product of the independent variable and its natural logarithm (after inclusion of the independent variable in the model). Three interaction terms had p-values of 0.046, 0.036 and 0.025, which do not survive correction for multiple testing of the three independent variables. A fourth interaction term had a lower p-value of 0.005 for regression of Narrow Neg on birthweight in the register twins. Adding a power term to the logistic regression analysis, birthweight^2^ was associated with Narrow Neg in the register twins (OR 3.41, 95%CI 1.42 to 8.15; p=0.006). However, birthweight^2^ was not associated with Narrow Neg in the non-register twins, nor in the combined samples analysis. In summary, there was evidence for a non-linear association between birthweight and Narrow Neg specifically in the register twins, but the conclusion from the main combined samples analysis remained that there was no significant association between birthweight and Narrow Neg.

Testing the fit of logistic regression models

We investigated the goodness-of-fit of logistic regression models using the Hosmer and Lemeshow test, where a good fit was indicated by a non-significant p-value (p>=0.05). For the logistic regression analysis of Narrow Pos on age at onset, adjusted for sex and illness duration, in the non-register twins there was a significant result (χ^2^=15.77, df=8, p=0.046), but this does not survive correction for multiple testing of pairs of symptom dimensions and independent variables, so we accepted the logistic regression analysis result.

Generalized linear mixed models additional investigation using robust estimation

For generalized linear mixed models showing associations in the combined samples, we re-ran analyses using the robust estimation option instead of the model-based estimation option, in case there were undetected violations of the model assumptions. All results remained the same, supporting the validity of the main analysis models.

Relationships between variables using non-parametric regression analysis

In order to further investigate the relationships between quantitative independent variables (age at onset, birthweight and premorbid IQ) and narrow symptom dimensions, we additionally conducted non-parametric regression analysis for binary dependent variables using the sm.binomial() function, which is part of the sm package in R (https://cran.r-project.org/package=sm). This function estimates a regression curve using a local likelihood approach, without the assumptions of the logistic regression model. First, we used the h.select([independent variable], [binary symptom dimension], method="aicc") function to select a smoothing parameter, then applied this in sm.binomial([independent variable], [binary symptom dimension], h=[value from h.select()]). Most output graphs were consistent with linear relationships between independent variables and the probability of narrow symptom dimensions. A possible exception was for birthweight and Narrow Neg in the register twin sample. This had a shallow u-shaped regression curve, with a minimum at 2.5kg (in the register twins with birthweight data n=107, mean 2.51kg (s.d. 0.68), range 1.14 to 4.09kg), which corroborates the evidence described above for a non-linear association between birthweight and Narrow Neg in the logistic regression analysis.

Statistical significance of associations in logistic regression analysis

Variables were described as associated where p<0.05, two-tailed. Due to the notable correlations between variables, a Bonferroni adjustment for multiple statistical tests would be over-conservative so, for the main results (Table 2) where there were 42 analyses of associations between the 3 narrow symptom dimensions and 14 clinical/developmental variables in the combined samples, we calculated the False Discovery Rate (FDR) using the Benjamini-Hochberg approach and used FDR=0.05 as the primary threshold for statistical significance. We have included the script as a Supplementary File

**References**

1. McGuffin P, Farmer A, Harvey I. A polydiagnostic application of operational criteria in studies of psychotic illness. Development and reliability of the OPCRIT system. *Arch Gen Psychiatry* 1991; **48**: 764-70.

2. Williams J, Farmer AE, Ackenheil M, Kaufmann CA, McGuffin P. A multicentre inter-rater reliability study using the OPCRIT computerized diagnostic system. *Psychol Med* 1996; **26**: 775-83.

3. Allardyce J, McCreadie RG, Morrison G, van Os J. Do symptom dimensions or categorical diagnoses best discriminate between known risk factors for psychosis? *Soc Psychiatry Psychiatr Epidemiol* 2007; **42**: 429-37.

4. Allardyce J, Morrison G, Van Os J, Kelly J, Murray RM, McCreadie RG. Schizophrenia is not disappearing in south-west Scotland. *Br J Psychiatry* 2000; **177**: 38-41.

5. Cardno AG, Marshall EJ, Coid B, Macdonald AM, Ribchester TR, Davies NJ, et al. Heritability estimates for psychotic disorders: the Maudsley twin psychosis series. *Arch Gen Psychiatry* 1999; **56**: 162-8.

6. Toulopoulou T, Picchioni M, Rijsdijk F, Hua-Hall M, Ettinger U, Sham P, et al. Substantial genetic overlap between neurocognition and schizophrenia: genetic modeling in twin samples. *Arch Gen Psychiatry* 2007; **64**: 1348-55.

7. Georgiades A, Rijsdijk F, Kane F, Rebollo-Mesa I, Kalidindi S, Schulze KK, et al. New insights into the endophenotypic status of cognition in bipolar disorder: genetic modelling study of twins and siblings. *Br J Psychiatry* 2016; **208**: 539-47.

8. Cardno AG, Sham PC, Murray RM, McGuffin P. Twin study of symptom dimensions in psychoses. *Br J Psychiatry* 2001; **179**: 39-45.

9. Cardno AG, Jones LA, Murphy KC, Sanders RD, Asherson P, Owen MJ, et al. Dimensions of psychosis in affected sibling pairs. *Schizophr Bull* 1999; **25**: 841-50.

10. Andreasen NC. *The Scale for the Assessment of Negative Symptoms (SANS).* Iowa City, IA: University of Iowa, 1984.

11. Andreasen NC. *The Scale for the Assessment of Positive Symptoms (SAPS).* Iowa City, IA: University of Iowa, 1984.

12. Liddle PF. The symptoms of chronic schizophrenia. A re-examination of the positive-negative dichotomy. *Br J Psychiatry* 1987; **151**: 145-51.

13. Andreasen NC, Arndt S, Alliger R, Miller D, Flaum M. Symptoms of schizophrenia. Methods, meanings, and mechanisms. *Arch Gen Psychiatry* 1995; **52**: 352-60.

14. Grube BS, Bilder RM, Goldman RS. Meta-analysis of symptom factors in schizophrenia. *Schizophr Res* 1998; **31**: 113-20.

15. Legge SE, Cardno AG, Allardyce J, Dennison C, Hubbard L, Pardiñas AF, et al. Associations Between Schizophrenia Polygenic Liability, Symptom Dimensions, and Cognitive Ability in Schizophrenia. *JAMA Psychiatry* 2021; **78**: 1143-51.

16. Kay SR, Fiszbein A, Opler LA. The positive and negative syndrome scale (PANSS) for schizophrenia. *Schizophr Bull* 1987; **13**: 261-76.

17. Rossi AC, Mullin PM, Chmait RH. Neonatal outcomes of twins according to birth order, presentation and mode of delivery: a systematic review and meta-analysis. *BJOG* 2011; **118**: 523-32.

18. Nelson HE. *National Adult Reading Test. Test Manual.* Windsor, UK: NFER-Nelson, 1982.

19. Rijsdijk FV, Gottesman II, McGuffin P, Cardno AG. Heritability estimates for psychotic symptom dimensions in twins with psychotic disorders. *Am J Med Genet Part B, Neuropsychiatr Genet* 2011; **156b**: 89-98.

20. Cardno AG, Rijsdijk FV, Murray RM, McGuffin P. Twin study refining psychotic symptom dimensions as phenotypes for genetic research. *Am J Med Genet Part B, Neuropsychiatr Genet* 2008; **147B**: 1213-21.

21. Toulopoulou T, Goldberg TE, Rebollo Mesa I, Picchioni M, Rijsdijk F, Stahl D, et al. Impaired intellect and memory: a missing link between genetic risk and schizophrenia? *Arch Gen Psychiatry* 2010; **67**: 905-13.

22. Owens SF, Picchioni MM, Ettinger U, McDonald C, Walshe M, Schmechtig A, et al. Prefrontal deviations in function but not volume are putative endophenotypes for schizophrenia. *Brain* 2012; **135**: 2231-44.
