# Supplementary material for: Associations of psychotic symptom dimensions with clinical and developmental variables in twin and general clinical samples
Source: Br J Psychiatry. 2025 Jan;226(1):16–23. doi: 10.1192/bjp.2024.129 (PMC11781858; doi:10.1192/bjp.2024.129)
Supplement: Cardno et al. supplementary material 3 — Cardno et al. supplementary material [file S0007125024001296sup003.docx]

**Associations of psychotic symptom dimensions with clinical and developmental variables in twin and general clinical samples**

Cardno AG, et al.

**Supplementary File - False Discovery Rate calculation in SPSS**

Benjamini-Hochberg approach, FDR=0.05.

Source:

Bruce Weaver, Lakehead University Thunder Bay Campus (28 Aug 2015)

https://www.researchgate.net/post/How_can_I_calculate_false_discovery_rate_using_spss. Accessed 29 March 2024.

Citation:

Weaver, Bruce. (2015). Re: How can I calculate false discovery rate using spss?. Retrieved from: https://www.researchgate.net/post/How_can_I_calculate_false_discovery_rate_using_spss/54dfa502d5a3f276158b469e/citation/download.

See also:

https://www.ibm.com/support/pages/does-spss-statistics-offer-multiple-comparisons-using-benjamini-hochberg-method-control-false-discovery-rate

Instructions:

1. In SPSS, click on **File > New > Syntax** to open a new syntax window.
2. Copy the following lines of syntax and paste them into the syntax window.
3. Enter the list of p-values between the BEGIN DATA and END DATA lines.
4. In the toolbar of the syntax window, click on **Run > All**.
5. View the results (of the LIST command) in the Output viewer.  Significant results will have variable test = 1.

DATA LIST free / p (F5.3).

BEGIN DATA

0.240 0.001 0.152 0.011 0.668 0.652 0.000029 0.000044 0.000003 0.000026 0.000008 0.001 0.052 0.867 0.246 0.007 0.301 0.026 0.091 0.166 0.773 0.000001 2.98E-7 2.65E-9 0.516 0.007 0.000428 6.93E-8 1.00E-14 9.44E-8 0.066 0.367 0.420 0.740 0.671 0.141 0.317 0.245 0.140 0.422 0.043 0.005

END DATA.

SORT CASES by p (a).

COMPUTE i=$casenum.

SORT CASES by i (d).

COMPUTE q=.05.

COMPUTE m=max(i,lag(m)).

COMPUTE crit=q*i/m.

COMPUTE test=(p le crit).

COMPUTE test=max(test,lag(test)).

FORMATS i m test(f8.0) q (f8.2) crit(f8.6).

VALUE LABELS test 1 'Significant' 0 'Not Significant'.

LIST.
