# Supplementary material for: Associations of psychotic symptom dimensions with clinical and developmental variables in twin and general clinical samples
Source: Br J Psychiatry. 2025 Jan;226(1):16–23. doi: 10.1192/bjp.2024.129 (PMC11781858; doi:10.1192/bjp.2024.129)
Supplement: Cardno et al. supplementary material 4 — Cardno et al. supplementary material [file S0007125024001296sup004.docx]

**Associations of psychotic symptom dimensions with clinical and developmental variables in twin and general clinical samples**

Cardno AG, et al.

**Supplementary Tables**

| Table S1. Probandwise concordance for diagnosis in the two twin samples | | | | |
| --- | --- | --- | --- | --- |
| Sample | Schizophrenia | | Any psychotic disorder^a^ | |
|  | MZ | DZ | MZ | DZ |
| Register twins (DSM-III-R)^b^ | 20/47 (42.6%) | 0/50 (0.0) | 60/106 (56.6%) | 12/118 (10.2%) |
| Non-register twins (DSM-IV) | 28/54 (51.9%) | 0/14 (0.0) | 50/85 (58.8%) | 4/27 (14.8%) |
|  |  |  |  |  |
| MZ, monozygotic; DZ, dizygotic.  a. Broadly defined to include disorders with any psychotic symptoms or manic/hypomanic episodes.  b. Schizophrenia concordance from Cardno et al (1999) and psychosis concordance from Rijsdijk et al (2010). | | | | |

| Table S2. Descriptive statistics for psychotic symptom dimensions in twin probands of monozygotic (MZ) and dizygotic (DZ) twin pairs | | | | | | | | | | |
| --- | --- | --- | --- | --- | --- | --- | --- | --- | --- | --- |
| Twin sample and zygosity | n | Positive dimension (0-2) | | | Negative dimension (0-2) | | | Disorganised dimension (0-2) | | |
|  |  | 0 | 1 | 2 | 0 | 1 | 2 | 0 | 1 | 2 |
| Register twins |  |  |  |  |  |  |  |  |  |  |
| MZ (%) | 106 | 4.7% | 29.2% | 66.0% | 50.9% | 37.7% | 11.3% | 46.2% | 31.1% | 22.6% |
| DZ (%) | 118 | 3.4% | 26.3% | 70.3% | 55.9% | 24.6% | 19.5% | 44.9% | 32.2% | 22.9% |
|  |  |  |  |  |  |  |  |  |  |  |
| Non-register twins |  |  |  |  |  |  |  |  |  |  |
| MZ (%) | 85 | 14.1% | 14.1% | 71.8% | 51.8% | 23.5% | 24.7% | 71.8% | 22.4% | 5.9% |
| DZ (%) | 27 | 11.1% | 29.6% | 59.3% | 63.0% | 29.6% | 7.4% | 74.1% | 22.2% | 3.7% |
|  |  |  |  |  |  |  |  |  |  |  |
| Combined samples |  |  |  |  |  |  |  |  |  |  |
| MZ (%) | 191 | 8.9% | 22.5% | 68.6% | 51.3% | 31.4% | 17.3% | 57.6% | 27.2% | 15.2% |
| DZ (%) | 145 | 4.8% | 26.9% | 68.3% | 57.2% | 25.5% | 17.2% | 50.3% | 30.3% | 19.3% |
|  |  |  |  |  |  |  |  |  |  |  |

| Table S3. Spearman intercorrelations between psychotic symptom dimensions (0-2 scores) | | | | |
| --- | --- | --- | --- | --- |
| Sample | Psychotic symptom dimensions (r_s_ (n)) | | | |
|  |  | Positive | Negative | Disorganised |
| Twin  (register) | Positive | - | 0.37*  (n=223) | 0.39*  (n=223) |
|  | Negative |  | - | 0.39*  (n=223) |
|  |  |  |  |  |
| Twin  (non-register) | Positive | - | 0.52*  (n=123) | 0.31*  (n=123) |
|  | Negative |  | - | 0.24*  (n=123) |
|  |  |  |  |  |
| CVPS | Positive | - | 0.28*  (n=75) | 0.10  (n=75) |
|  | Negative |  | - | 0.12  (n=75) |
|  |  |  |  |  |
| D&G | Positive | - | 0.15*  (n=456) | 0.13*  (n=456) |
|  | Negative |  | - | 0.41*  (n=456) |
| CVPS, Clinical Variation in Psychoses Study; D&G, Dumfries and Galloway Psychosis Study.  *Statistically significant at p<0.05, two-tailed. | | | | |

| Table S4. Spearman correlations between psychotic symptom dimensions and the overall balance of psychotic and affective symptoms^a^ | | | | |
| --- | --- | --- | --- | --- |
| Sample | n | Positive dimension (0-2) | Negative dimension (0-2) | Disorganised dimension (0-2) |
|  |  |  |  |  |
| Twin  (register) | 202 | -0.40* | -0.40* | -0.37* |
| Twin  (non-register) | 79 | -0.64* | -0.85* | -0.56* |
| CVPS | 57 | -0.58* | -0.53* | -0.08 |
| D&G | 267 | -0.39* | -0.40* | -0.22* |
| CVPS, Clinical Variation in Psychoses Study; D&G, Dumfries and Galloway Psychosis Study.  a. Balance of psychotic and affective symptoms from OPCRIT item 52: negative correlation indicates association with psychotic symptoms predominating, and positive correlation indicates association with affective symptoms predominating (cases with score 0 – no co-occurrence – excluded).  *Statistically significant at p<0.05, two-tailed. | | | | |

| Table S5. Logistic regression analysis of psychotic symptom dimensions on demographic, developmental and clinical variables in the individual and combined samples – adjusted for sex and illness duration^a^ | | | | | | | | | | | | |
| --- | --- | --- | --- | --- | --- | --- | --- | --- | --- | --- | --- | --- |
| Independent variable | Sample | n | Positive dimension | | Negative dimension | | | | Disorganised dimension | | | |
|  |  |  | narrow (0-1 vs 2) | | broad (0 vs 1-2) | | narrow (0-1 vs 2) | | broad (0 vs 1-2) | | narrow (0-1 vs 2) | |
|  |  |  | OR (95% CI) | p-value | OR (95% CI) | p-value | OR (95% CI) | p-value | OR (95% CI) | p-value | OR (95% CI) | p-value |
| Male sex  [this analysis not adjusted for sex] | Twin (register) | 223 | 0.84 (0.47-1.49) | 0.551 | 1.16 (0.68-1.98) | 0.591 | 1.01 (0.49-2.12) | 0.971 | 1.09 (0.64-1.86) | 0.752 | 1.22 (0.64-2.31) | 0.543 |
|  | Twin  (non-reg.) | 121 | 2.19 (0.96-4.97) | 0.062 | 3.99 (1.81-8.77) | 0.001* | 4.82 (1.64-14.14) | 0.004* | 0.92 (0.41-2.03) | 0.828 | 0.15 (0.02-1.26) | 0.080 |
|  | CVPS | 75 | 0.61 (0.18-2.05) | 0.428 | NC |  | NC |  | 5.45 (0.66-44.85) | 0.115 | NC |  |
|  | D&G | 456 | 1.27 (0.88-1.85) | 0.208 | 2.08 (1.34-3.22) | 0.001* | 2.32 (1.23-4.36) | 0.009* | 1.67 (1.14-2.45) | 0.009* | 1.68 (0.99-2.86) | 0.054 |
|  | Combined | 875 | 1.18 (0.90-1.56) | 0.240 | 1.95 (1.44-2.63) | 0.000015* | 2.02 (1.32-3.09) | 0.001* | 1.42 (1.07-1.88) | 0.014* | 1.33 (0.90-1.95) | 0.152 |
|  |  |  |  |  |  |  |  |  |  |  |  |  |
| Non-white ethnicity | Twin (register) | 223 | 3.20 (1.04-9.83) | 0.043* | 1.41 (0.61-3.25) | 0.416 | 1.59 (0.57-4.43) | 0.376 | 1.79 (0.75-4.27) | 0.191 | 0.98 (0.36-2.64) | 0.960 |
|  | Twin  (non-reg.) | 114 | 0.88 (0.08-9.28) | 0.913 | 3.70 (0.33-42.02) | 0.291 | 1.04 (0.09-12.21) | 0.977 | 0.82 (0.08-8.48) | 0.871 | NC |  |
|  | CVPS | 75 | 6.47 (1.26-33.20) | 0.025* | 1.71 (0.40-7.29) | 0.469 | NC |  | 0.37 (0.07-1.95) | 0.240 | NC |  |
|  | D&G |  | NA |  | NA |  | NA |  | NA |  | NA |  |
|  | Combined | 412 | 3.07 (1.30-7.25) | 0.011* | 1.58 (0.80-3.15) | 0.191 | 1.22 (0.49-3.06) | 0.668 | 1.21 (0.62-2.39) | 0.576 | 0.80 (0.31-2.10) | 0.652 |
|  |  |  |  |  |  |  |  |  |  |  |  |  |
| Poor premorbid social adjustment | Twin (register) | 223 | 2.05 (1.05-4.01) | 0.037* | 1.78 (0.99-3.21) | 0.054 | 1.49 (0.69-3.20) | 0.311 | 2.40 (1.30-4.42) | 0.005* | 2.36 (1.22-4.57) | 0.011* |
|  | Twin  (non-reg.) | 121 | 3.64 (1.22-10.86) | 0.020* | 4.48 (1.75-11.46) | 0.002* | 1.83 (0.66-5.08) | 0.243 | 2.32 (0.99-5.44) | 0.052 | 1.04 (0.18-5.90) | 0.967 |
|  | CVPS | 75 | 1.98 (0.53-7.34) | 0.310 | 4.46 (1.05-19.06) | 0.043* | NC |  | 2.22 (0.60-8.29) | 0.234 | NC |  |
|  | D&G | 456 | 1.77 (1.16-2.68) | 0.008* | 3.93 (2.47-6.26) | 8.397E-9* | 3.42 (1.80-6.50) | 0.000171* | 2.61 (1.71-3.99) | 0.000010* | 2.83 (1.64-4.88) | 0.000192* |
|  | Combined | 875 | 1.99 (1.44-2.74) | 0.000029* | 3.05 (2.21-4.22) | 1.934E-11* | 2.41 (1.58-3.68) | 0.000044* | 2.52 (1.85-3.43) | 6.369E-9* | 2.57 (1.73-3.81) | 0.000003* |
|  |  |  |  |  |  |  |  |  |  |  |  |  |
| Never married or cohabited | Twin (register) | 223 | 3.29 (1.78-6.07) | 0.000139* | 1.55 (0.90-2.69) | 0.115 | 1.82 (0.84-3.96) | 0.130 | 2.79 (1.59-4.90) | 0.000356* | 1.92 (0.98-3.77) | 0.056 |
|  | Twin  (non-reg.) | 121 | 6.64 (2.58-17.11) | 0.000088* | 4.81 (1.72-13.47) | 0.003* | 12.09 (1.48-98.87) | 0.020* | 11.73 (2.90-47.42) | 0.001* | 4.18 (0.39-45.07) | 0.238 |
|  | CVPS | 75 | 2.26 (0.84-6.06) | 0.105 | 1.56 (0.43-5.71) | 0.499 | NC |  | 3.43 (1.07-11.03) | 0.038* | NC |  |
|  | D&G | 456 | 0.95 (0.64-1.40) | 0.794 | 1.67 (1.07-2.62) | 0.025* | 2.25 (1.17-4.31) | 0.015* | 1.70 (1.14-2.53) | 0.009* | 1.83 (1.06-3.16) | 0.030* |
|  | Combined | 875 | 1.85 (1.39-2.45) | 0.000026* | 2.05 (1.51-2.79) | 0.000006* | 2.86 (1.81-4.53) | 0.000008* | 2.51 (1.87-3.36) | 1.0066E-9* | 2.03 (1.36-3.03) | 0.001* |
|  |  |  |  |  |  |  |  |  |  |  |  |  |
| Premorbid drug/alcohol abuse (within year prior to onset) | Twin (register) | 223 | 2.27 (0.80-6.38) | 0.122 | 1.29 (0.56-2.99) | 0.545 | 0.93 (0.29-2.95) | 0.901 | 0.94 (0.41-2.17) | 0.880 | 0.99 (0.37-2.66) | 0.983 |
|  | Twin  (non-reg.) | 121 | NC |  | 0.61 (0.11-3.49) | 0.582 | 1.09 (0.17-6.77) | 0.930 | 0.45 (0.05-4.08) | 0.475 | 9.84 (0.54-178.13) | 0.122 |
|  | CVPS | 75 | 0.99 (0.37-2.67) | 0.989 | 0.49 (0.13-1.83) | 0.288 | NC |  | 0.59 (0.19-1.87) | 0.369 | NC |  |
|  | D&G | 456 | 1.49 (0.89-2.47) | 0.127 | 1.45 (0.83-2.51) | 0.191 | 1.20 (0.56-2.59) | 0.636 | 1.87 (1.12-3.12) | 0.016* | 1.38 (0.71-2.67) | 0.343 |
|  | Combined | 875 | 1.48 (1.00-2.19) | 0.052 | 1.17 (0.78-1.77) | 0.446 | 0.95 (0.53-1.71) | 0.867 | 1.34 (0.91-1.97) | 0.134 | 1.35 (0.81-2.26) | 0.246 |
|  |  |  |  |  |  |  |  |  |  |  |  |  |
| Lifetime cannabis abuse/regular use | Twin (register) | 223 | 6.79 (0.85-54.50) | 0.071 | 2.51 (0.73-8.69) | 0.146 | 3.84 (1.10-13.39) | 0.035* | 0.91 (0.29-2.90) | 0.873 | 3.44 (1.04-11.35) | 0.043* |
|  | Twin  (non-reg.) | 121 | 0.50 (0.11-2.28) | 0.372 | 0.39 (0.09-1.77) | 0.223 | 0.33 (0.04-2.95) | 0.324 | 0.62 (0.12-3.24) | 0.571 | 2.14 (0.18-25.22) | 0.545 |
|  | CVPS | 75 | 0.93 (0.34-2.50) | 0.879 | 0.54 (0.15-1.98) | 0.350 | NC |  | 1.18 (0.39-3.59) | 0.766 | NC |  |
|  | D&G | 456 | 2.75 (1.43-5.29) | 0.002* | 1.49 (0.79-2.80) | 0.217 | 1.66 (0.73-3.78) | 0.231 | 2.37 (1.30-4.34) | 0.005* | 1.42 (0.67-3.02) | 0.356 |
|  | Combined | 875 | 1.89 (1.19-3.00) | 0.007* | 1.25 (0.78-1.99) | 0.358 | 1.39 (0.74-2.61) | 0.301 | 1.70 (1.09-2.66) | 0.019* | 1.92 (1.08-3.40) | 0.026* |
|  |  |  |  |  |  |  |  |  |  |  |  |  |
| Psychosocial precipitant (within 6 months prior to onset) | Twin (register) | 223 | 0.51 (0.28-0.92) | 0.025* | 0.53 (0.30-0.93) | 0.028* | 0.64 (0.28-1.45) | 0.286 | 0.69 (0.39-1.21) | 0.194 | 0.70 (0.35-1.40) | 0.315 |
|  | Twin  (non-reg.) | 121 | 1.51 (0.32-7.01) | 0.601 | 1.36 (0.32-5.79) | 0.682 | 3.92 (0.72-21.33) | 0.114 | 1.70 (0.44-6.60) | 0.447 | 3.58 (0.56-22.69) | 0.177 |
|  | CVPS | 75 | 1.65 (0.64-4.27) | 0.300 | 2.56 (0.69-9.48) | 0.161 | NC |  | 1.08 (0.36-3.22) | 0.891 | NC |  |
|  | D&G | 456 | 0.71 (0.44-1.15) | 0.165 | 0.43 (0.22-0.85) | 0.014* | 0.51 (0.19-1.34) | 0.171 | 0.81 (0.49-1.34) | 0.406 | 1.05 (0.53-2.08) | 0.880 |
|  | Combined | 875 | 0.75 (0.54-1.05) | 0.091 | 0.62 (0.42-0.91) | 0.014* | 0.67 (0.38-1.18) | 0.166 | 0.84 (0.60-1.18) | 0.320 | 0.93 (0.59-1.49) | 0.773 |
|  |  |  |  |  |  |  |  |  |  |  |  |  |
| Age at onset (first contact with mental health services) | Twin (register) | 223 | 0.95 (0.92-0.97) | 0.000076* | 0.96 (0.94-0.99) | 0.004* | 0.94 (0.90-0.99) | 0.015* | 0.93 (0.91-0.96) | 0.000008* | 0.95 (0.91-0.99) | 0.010* |
|  | Twin  (non-reg.) | 121 | 0.97 (0.91-1.03) | 0.330 | 1.02 (0.95-1.08) | 0.628 | 0.96 (0.88-1.05) | 0.416 | 0.99 (0.92-1.05) | 0.677 | 0.97 (0.85-1.11) | 0.639 |
|  | CVPS | 75 | 0.94 (0.88-0.99) | 0.031* | 0.87 (0.76-0.98) | 0.027* | NC |  | 0.94 (0.87-1.01) | 0.102 | NC |  |
|  | D&G | 456 | 0.99 (0.98-1.00) | 0.023* | 0.96 (0.94-0.97) | 1.015E-7* | 0.94 (0.91-0.97) | 0.000070* | 0.95 (0.94-0.96) | 5.553E-13* | 0.94 (0.92-0.96) | 0.000001* |
|  | Combined | 875 | 0.98 (0.97-0.99) | 0.000001* | 0.96 (0.94-0.97) | 1.013E-11* | 0.94 (0.92-0.96) | 2.98E-7* | 0.95 (0.94-0.96) | <1.00E-14* | 0.94 (0.92-0.96) | 2.65E-9* |
|  |  |  |  |  |  |  |  |  |  |  |  |  |
| Gradual onset (over more than 6 months) | Twin (register) | 196 | 1.92 (1.03-3.56) | 0.040* | 1.77 (0.99-3.18) | 0.054 | 2.17 (0.94-5.02) | 0.071 | 1.72 (0.96-3.06) | 0.067 | 2.20 (1.07-4.54) | 0.033* |
|  | Twin  (non-reg.) | 48 | 3.92 (0.35-43.52) | 0.265 | 5.30 (0.77-36.69) | 0.091 | 0.83 (0.12-5.61) | 0.849 | 1.40 (0.31-6.37) | 0.662 | 0.73 (0.06-9.31) | 0.811 |
|  | CVPS | 37 | 11.60 (1.80-74.71) | 0.010* | NC |  | NC |  | 0.17 (0.02-1.67) | 0.127 | NC |  |
|  | D&G | 452 | 0.80 (0.54-1.18) | 0.249 | 2.10 (1.33-3.33) | 0.002* | 2.65 (1.40-5.00) | 0.003* | 1.44 (0.96-2.16) | 0.082 | 2.54 (1.46-4.41) | 0.001* |
|  | Combined | 733 | 1.11 (0.81-1.52) | 0.516 | 1.88 (1.34-2.64) | 0.000266* | 1.89 (1.19-3.01) | 0.007* | 1.32 (0.97-1.80) | 0.080 | 2.05 (1.38-3.05) | 0.000428* |
|  |  |  |  |  |  |  |  |  |  |  |  |  |
| Chronic illness course | Twin (register) | 218 | 5.08 (2.26-11.44) | 0.000087* | 4.51 (2.41-8.47) | 0.000003* | 5.03 (2.33-10.85) | 0.000039* | 4.48 (2.31-8.67) | 0.000009* | 5.02 (2.55-9.87) | 0.000003* |
|  | Twin  (non-reg.) | 94 | 25.92 (3.23-208.35) | 0.002* | 22.84 (6.64-78.53) | 6.856E-7* | 30.29 (5.75-159.56) | 0.000058* | 4.02 (1.62-10.01) | 0.003* | 6.45 (1.09-38.12) | 0.040* |
|  | CVPS | 75 | 4.00 (0.94-17.08) | 0.062 | 2.03 (0.41-10.00) | 0.383 | NC |  | 1.40 (0.36-5.51) | 0.631 | NC |  |
|  | D&G | 455 | 1.57 (1.06-2.32) | 0.024* | 6.52 (4.01-10.59) | 3.822E-14* | 11.59 (5.19-25.89) | 2.248E-9* | 1.81 (1.21-2.71) | 0.004* | 2.15 (1.26-3.67) | 0.005* |
|  | Combined | 842 | 2.41 (1.76-3.31) | 6.93E-8* | 5.74 (4.10-8.05) | <1.00E-14* | 9.06 (5.52-14.89) | <1.00E-14* | 2.37 (1.76-3.20) | 2.31E-8* | 2.95 (1.99-4.37) | 9.44E-8* |
|  |  |  |  |  |  |  |  |  |  |  |  |  |
| Birth order (being second born in twin pair)  [twins only] | Twin (register) | 193 | 0.63 (0.34-1.17) | 0.143 | 0.76 (0.43-1.35) | 0.350 | 0.85 (0.40-1.80) | 0.674 | 0.64 (0.36-1.13) | 0.122 | 1.13 (0.59-2.16) | 0.724 |
|  | Twin  (non-reg.) | 59 | 0.24 (0.02-2.43) | 0.226 | 0.44 (0.13-1.44) | 0.174 | 0.63 (0.20-2.00) | 0.434 | 3.30 (1.00-10.90) | 0.050 | 3.51 (0.31-40.17) | 0.313 |
|  | CVPS |  |  |  |  |  |  |  |  |  |  |  |
|  | D&G |  |  |  |  |  |  |  |  |  |  |  |
|  | Combined | 252 | 0.58 (0.32-1.04) | 0.066 | 0.67 (0.40-1.11) | 0.120 | 0.75 (0.41-1.40) | 0.367 | 0.86 (0.52-1.43) | 0.567 | 1.29 (0.69-2.39) | 0.420 |
|  |  |  |  |  |  |  |  |  |  |  |  |  |
| Birthweight (kg) | Twin (register) | 107 | 1.49 (0.77-2.89) | 0.242 | 0.75 (0.42-1.34) | 0.332 | 0.95 (0.47-1.93) | 0.888 | 1.18 (0.65-2.13) | 0.594 | 2.08 (1.02-4.24) | 0.044* |
|  | Twin  (non-reg.) | 55 | 0.16 (0.02-1.10) | 0.062 | 1.53 (0.74-3.15) | 0.252 | 1.37 (0.67-2.79) | 0.389 | 0.85 (0.44-1.65) | 0.636 | 0.79 (0.24-2.61) | 0.702 |
|  | CVPS | 26 | 1.57 (0.55-4.45) | 0.400 | 1.82 (0.38-8.60) | 0.452 | NC |  | 1.03 (0.35-3.04) | 0.955 | NC |  |
|  | D&G |  |  |  |  |  |  |  |  |  |  |  |
|  | Combined | 188 | 1.08 (0.68-1.74) | 0.740 | 1.02 (0.68-1.54) | 0.099 | 1.11 (0.68-1.81) | 0.671 | 1.02 (0.69-1.50) | 0.073 | 1.51 (0.87-2.61) | 0.141 |
|  |  |  |  |  |  |  |  |  |  |  |  |  |
| Handedness (being non-right handed) | Twin (register) | 129 | 2.05 (0.64-6.54) | 0.226 | 4.58 (1.57-13.37) | 0.005* | 1.73 (0.55-5.45) | 0.346 | 1.19 (0.47-3.00) | 0.717 | 1.51 (0.56-4.13) | 0.418 |
|  | Twin  (non-reg.) | 33 | NC |  | 1.04 (0.05-21.04) | 0.978 | 12.70 (0.38-421.91) | 0.155 | NC |  | NC |  |
|  | CVPS | 47 | 0.81 (0.10-6.59) | 0.847 | NC |  | NC |  | NC |  | NC |  |
|  | D&G |  |  |  |  |  |  |  |  |  |  |  |
|  | Combined | 209 | 1.65 (0.62-4.43) | 0.317 | 2.79 (1.17-6.64) | 0.021* | 1.86 (0.65-5.32) | 0.245 | 1.16 (0.51-2.64) | 0.723 | 1.97 (0.80-4.88) | 0.140 |
|  |  |  |  |  |  |  |  |  |  |  |  |  |
| Premorbid IQ (based on NART)  [twins only] | Twin (register) | 46 | 1.00 (0.94-1.06) | 0.930 | 0.99 (0.94-1.04) | 0.723 | 0.97 (0.90-1.04) | 0.342 | 0.93 (0.87-0.99) | 0.021* | 0.93 (0.87-0.99) | 0.025* |
|  | Twin  (non-reg.) | 37 | 0.78 (0.51-1.19) | 0.250 | 0.92 (0.85-1.00) | 0.057 | 0.94 (0.88-0.995) | 0.035* | 0.92 (0.87-0.98) | 0.014* | 0.93 (0.84-1.03) | 0.156 |
|  | CVPS |  |  |  |  |  |  |  |  |  |  |  |
|  | D&G |  |  |  |  |  |  |  |  |  |  |  |
|  | Combined | 83 | 0.98 (0.93-1.03) | 0.422 | 0.97 (0.93-1.01) | 0.118 | 0.96 (0.92-1.00) | 0.043* | 0.93 (0.89-0.97) | 0.001* | 0.92 (0.88-0.98) | 0.005* |
|  |  |  |  |  |  |  |  |  |  |  |  |  |
| OR, odds ratio; 95% CI, 95% confidence interval; CVPS, Clinical Variation in Psychoses Study; D&G, Dumfries and Galloway Psychosis Study; NART, National Adult Reading Test; NC, Not calculated for individual sample due to insufficient variation in data, but included in combined analysis; NA, Not applicable because no participant in sample of non-white ethnicity.  a. Combined analysis used generalized linear mixed model with addition of sample modelled as a random effect.  *Statistically significant at p<0.05, two-tailed. | | | | | | | | | | | | |

| Table S6. Descriptive statistics for psychotic symptom dimensions and demographic, developmental and clinical variables among probands in the combined samples | | | | | | | | | | |
| --- | --- | --- | --- | --- | --- | --- | --- | --- | --- | --- |
| Demographic, developmental or clinical variable | n | Positive dimension (0-2) | | | Negative dimension (0-2) | | | Disorganised dimension (0-2) | | |
|  |  | 0 | 1 | 2 | 0 | 1 | 2 | 0 | 1 | 2 |
|  |  |  |  |  |  |  |  |  |  |  |
| Female sex (%) | 432 | 9.3% | 33.6% | 57.2% | 74.3% | 16.7% | 9.0% | 61.3% | 25.0% | 13.7% |
| Male sex (%) | 445 | 6.3% | 32.4% | 61.3% | 61.1% | 23.4% | 15.5% | 55.3% | 29.0% | 15.7% |
|  |  |  |  |  |  |  |  |  |  |  |
| White ethnicity (%) | 370 | 8.9% | 28.9% | 62.2% | 61.6% | 24.3% | 14.1% | 59.5% | 25.9% | 14.6% |
| Non-white ethnicity (%) | 44 | 2.3% | 13.6% | 84.1% | 50.0% | 34.1% | 15.9% | 52.3% | 34.1% | 13.6% |
|  |  |  |  |  |  |  |  |  |  |  |
| Not poor premorbid social adjustment (%) | 625 | 9.6% | 35.7% | 54.7% | 75.0% | 16.0% | 9.0% | 65.1% | 24.2% | 10.7% |
| Poor premorbid social adjustment (%) | 252 | 3.2% | 26.2% | 70.6% | 49.2% | 30.2% | 20.6% | 41.3% | 34.1% | 24.6% |
|  |  |  |  |  |  |  |  |  |  |  |
| Married or cohabited (%) | 454 | 10.6% | 37.9% | 51.5% | 76.4% | 17.0% | 6.6% | 67.8% | 21.1% | 11.0% |
| Never married or cohabited (%) | 423 | 4.7% | 27.7% | 67.6% | 58.2% | 23.4% | 18.4% | 48.0% | 33.3% | 18.7% |
|  |  |  |  |  |  |  |  |  |  |  |
| Not premorbid drug/alcohol abuse (%) | 729 | 8.8% | 33.2% | 58.0% | 67.9% | 19.6% | 12.5% | 59.3% | 26.5% | 14.3% |
| Premorbid drug/alcohol abuse (%) | 148 | 2.7% | 31.8% | 65.5% | 66.2% | 22.3% | 11.5% | 53.4% | 29.7% | 16.9% |
|  |  |  |  |  |  |  |  |  |  |  |
| Not lifetime cannabis abuse/regular use (%) | 770 | 8.3% | 33.9% | 57.8% | 67.8% | 20.1% | 12.1% | 59.2% | 26.6% | 14.2% |
| Lifetime cannabis abuse/regular use (%) | 107 | 3.7% | 26.2% | 70.1% | 66.4% | 19.6% | 14.0% | 51.4% | 29.9% | 18.7% |
|  |  |  |  |  |  |  |  |  |  |  |
| Not psychosocial precipitant (%) | 676 | 8.0% | 31.4% | 60.7% | 65.4% | 21.2% | 13.5% | 57.7% | 27.7% | 14.6% |
| Psychosocial  precipitant (%) | 201 | 7.0% | 38.3% | 54.7% | 75.1% | 16.4% | 8.5% | 60.2% | 24.9% | 14.9% |
|  |  |  |  |  |  |  |  |  |  |  |
| Age at onset (years)  (n, mean, (sd)) | 875 | 67  36.0 yrs  (21.2) | 288  38.3 yrs  (19.7) | 520  30.8 yrs  (16.3) | 592  37.3 yrs  (19.9) | 175  27.3 yrs  (11.6) | 108  24.1 yrs  (7.8) | 509  38.1 yrs  (20.6) | 237  28.7 yrs  (13.0) | 129  25.2 yrs  (8.9) |
|  |  |  |  |  |  |  |  |  |  |  |
| Not gradual onset (%) | 436 | 7.8% | 33.7% | 58.5% | 72.7% | 16.7% | 10.6% | 59.6% | 28.2% | 12.2% |
| Gradual onset (%) | 297 | 4.4% | 33.3% | 62.3% | 58.2% | 24.9% | 16.8% | 50.5% | 26.6% | 22.9% |
|  |  |  |  |  |  |  |  |  |  |  |
| Not chronic illness course (%) | 560 | 8.4% | 38.0% | 53.6% | 79.3% | 16.4% | 4.3% | 63.9% | 25.5% | 10.5% |
| Chronic illness  course (%) | 283 | 3.9% | 23.3% | 72.8% | 43.1% | 28.6% | 28.3% | 43.5% | 31.8% | 24.7% |
|  |  |  |  |  |  |  |  |  |  |  |
| First born twin (%) | 130 | 4.6% | 16.2% | 79.2% | 41.5% | 35.4% | 23.1% | 43.8% | 36.9% | 19.2% |
| Second born twin (%)^a^ | 122 | 2.5% | 27.9% | 69.7% | 50.8% | 30.3% | 18.9% | 47.5% | 29.5% | 23.0% |
|  |  |  |  |  |  |  |  |  |  |  |
| Birthweight (kg)  (n, mean, (sd)) | 188 | 7  3.1 kg  (0.6) | 40  2.7 kg  (0.8) | 141  2.5 kg  (0.8) | 84  2.7 kg  (0.9) | 64  2.5 kg  (0.8) | 40  2.5 kg  (0.8) | 88  2.6 kg  (0.9) | 66  2.5 kg  (0.8) | 34  2.7 kg  (0.8) |
|  |  |  |  |  |  |  |  |  |  |  |
| Right handed (%) | 180 | 4.4% | 26.1% | 69.4% | 57.8% | 28.3% | 13.9% | 53.3% | 30.0% | 16.7% |
| Non-right handed (%) | 29 | 3.4% | 17.2% | 79.3% | 34.5% | 44.8% | 20.7% | 44.8% | 24.1% | 31.0% |
|  |  |  |  |  |  |  |  |  |  |  |
| Premorbid IQ (based on NART) (n, mean, (sd)) | 83 | 0  -  - | 14  109.1 (11.2) | 69  103.9  (14.0) | 30  108.4  (12.2) | 31  105.2  (13.1) | 22  99.1  (14.8) | 38  109.7  (10.8) | 30  101.9  (14.9) | 15  97.9  (13.7) |
| yrs, years; sd, standard deviation; NART, National Adult Reading Test.  a. Includes one 3^rd^ born triplet. | | | | | | | | | | |

| Table S7. Logistic regression analysis of narrow psychotic symptom dimensions on demographic, developmental and clinical variables among probands in the combined samples – including the other symptom dimensions as covariates^a^ | | | | | | | |
| --- | --- | --- | --- | --- | --- | --- | --- |
| Independent variable | n | Positive dimension | | Negative dimension | | Disorganised dimension | |
|  |  | OR (95% CI) | p-value | OR (95% CI) | p-value | OR (95% CI) | p-value |
| Male sex  [this analysis not adjusted for sex] | 875 | 1.08 (0.81 to 1.43) | 0.606 | 1.93 (1.23 to 3.02) | 0.004* | 1.15 (0.77 to 1.74) | 0.496 |
| Non-white ethnicity  [D&G excluded as all White ethnicity] | 412 | 3.27 (1.38 to 7.79) | 0.007* | 1.00 (0.38 to 2.60) | 0.993 | 0.58 (0.22 to 1.59) | 0.292 |
| Poor premorbid social adjustment | 875 | 1.67 (1.20 to 2.33) | 0.002* | 1.76 (1.12 to 2.75) | 0.014* | 2.03 (1.33 to 3.09) | 0.000965* |
| Never married or cohabited | 875 | 1.61 (1.20 to 2.16) | 0.00140* | 2.22 (1.37 to 3.61) | 0.00134* | 1.59 (1.03 to 2.45) | 0.035* |
| Premorbid drug/alcohol abuse (within year prior to onset) | 875 | 1.46 (0.98 to 2.19) | 0.066 | 0.79 (0.43 to 1.46) | 0.445 | 1.28 (0.75 to 2.20) | 0.370 |
| Lifetime cannabis abuse/regular use | 875 | 1.79 (1.11 to 2.87) | 0.017* | 1.02 (0.53 to 1.97) | 0.955 | 1.49 (0.81 to 2.75) | 0.198 |
| Psychosocial precipitant (within 6 months prior to onset) | 875 | 0.78 (0.55 to 1.09) | 0.139 | 0.67 (0.37 to 1.22) | 0.194 | 1.13 (0.69 to 1.85) | 0.617 |
| Age at onset (first contact with mental health services) | 875 | 0.98 (0.96 to 0.99) | 0.000432* | 0.95 (0.93 to 0.98) | 0.000105* | 0.95 (0.93 to 0.97) | 0.00000152* |
| Gradual onset (over more than 6 months) | 733 | 0.99 (0.72 to 1.36) | 0.934 | 1.53 (0.94 to 2.52) | 0.090 | 1.85 (1.20 to 2.84) | 0.005* |
| Chronic illness course | 842 | 1.92 (1.37 to 2.69) | 0.000152* | 6.68 (4.01 to 11.13) | 6.102E-13* | 1.79 (1.16 to 2.77) | 0.009* |
| Birth order (being second born in twin pair)  [twins only] | 252 | 0.54 (0.29 to 1.01) | 0.052 | 0.76 (0.39 to 1.47) | 0.414 | 1.55 (0.79 to 3.02) | 0.199 |
| Birthweight (kg)  [D&G excluded as no information] | 188 | 0.99 (0.61 to 1.63) | 0.982 | 1.04 (0.63 to 1.70) | 0.889 | 1.50 (0.86 to 2.62) | 0.152 |
| Handedness (being non-right handed)  [D&G excluded as no information] | 209 | 1.42 (0.51 to 3.97) | 0.504 | 1.46 (0.49 to 4.37) | 0.498 | 1.75 (0.68 to 4.47) | 0.243 |
| Premorbid IQ (NART)  [twins only] | 83 | 1.01 (0.95 to 1.06) | 0.860 | 0.97 (0.93 to 1.01) | 0.141 | 0.92 (0.87 to 0.98) | 0.011* |
| OR, odds ratio; 95% CI, 95% confidence interval; D&G, Dumfries and Galloway Psychosis Study; NART, National Adult Reading Test.  a. Combined sample analysis used generalized linear mixed model, adjusted for sex, illness duration and the other two narrow symptom dimensions, with sample modelled as a random effect.  *Statistically significant at p<0.05, two-tailed. | | | | | | | |

| Table S8. Intraclass correlations of premorbid IQ (based on NART) within monozygotic (MZ) twin pairs concordant for any psychotic disorder. | | |
| --- | --- | --- |
| Sample | No. pairs | Intraclass correlation (95% CI) |
|  |  |  |
| Twin  (register) | 8 | 0.92 (0.65 to 0.98)* |
| Twin  (non-register) | 16 | 0.73 (0.38 to 0.90)* |
| Combined | 24 | 0.79 (0.58 to 0.90)* |
|  |  |  |
| NART, National Adult Reading Test; 95% CI, 95% confidence interval.  *Correlations with 95% CI not including zero treated as statistically significant at p<0.05, two-tailed. | | |

| Table S9. Logistic regression analysis of disorganised symptom dimension in twin probands on premorbid IQ (based on NART) in co-twins in the individual and combined twin samples – adjusted for sex and illness duration^a^ | | | | | |
| --- | --- | --- | --- | --- | --- |
| Sample | No. pairs | Disorganised dimension | | | |
|  |  | broad (0 vs 1-2) | | narrow (0-1 vs 2) | |
|  |  | OR (95% CI) | p-value | OR (95% CI) | p-value |
| MZ twin pairs |  |  |  |  |  |
| Twin  (register) | 26 | 0.91 (0.83 to 1.00) | 0.059 | 0.50 (0.22 to 1.17) | 0.109 |
| Twin  (non-register) | 29 | 0.96 (0.87 to 1.05) | 0.342 | 0.89 (0.73 to 1.09) | 0.267 |
| Combined | 55 | 0.95 (0.90 to 1.00) | 0.042* | 0.84 (0.71 to 0.99) | 0.039* |
|  |  |  |  |  |  |
| DZ twin pairs |  |  |  |  |  |
| Twin  (register) | 16 | 1.00 (0.85 to 1.16) | 0.953 | 0.86 (0.66 to 1.13) | 0.275 |
| Twin  (non-register) | 5 | NC |  | NC |  |
| Combined | 21 | 1.05 (0.91 to 1.21) | 0.528 | 1.03 (0.87 to 1.21) | 0.309 |
|  |  |  |  |  |  |
| NART, National Adult Reading Test; MZ, monozygotic; DZ, dizygotic; NC, not calculated due to insufficient variation in sample but included in combined analysis.  a. Combined analysis used generalized linear mixed model with addition of sample modelled as a random effect.  *Statistically significant at p<0.05, two-tailed. | | | | | |
